# CMV Encephalitis/Radiculitis: The Difficulty in Diagnosing in an Intubated Patient

**DOI:** 10.1155/2019/8067648

**Published:** 2019-02-18

**Authors:** Geoffrey Newcomb, Peter Mariuz, Daniel Lachant

**Affiliations:** ^1^Department of Internal Medicine, University of Rochester Medical Center, USA; ^2^Division of Infectious Diseases, University of Rochester Medical Center, USA; ^3^Division of Pulmonary and Critical Care Medicine, University of Rochester Medical Center, USA

## Abstract

Cytomegalovirus (CMV) can cause severe disease including colitis, pneumonitis, and less commonly encephalitis, in profoundly immunocompromised individuals. CNS imaging findings are nonspecific and diagnosis is made by identifying CMV in cerebral spinal fluid through PCR testing or cell culture. Early initiation of antiviral therapy is key with an overall poor outcome. Here we present a patient with newly diagnosed AIDS and* pneumocystis jiroveci *pneumonia who was febrile and remained encephalopathic for the first 6 weeks of his admission despite treatment and extensive work up for encephalopathy. Ultimately, he was diagnosed with CMV encephalitis and radiculitis and failed to improve significantly. This case is important because of multiple points (1) the uncommon presentation of CMV encephalitis/radiculitis occurring over 1 month into a hospitalization; (2) in the era of highly active antiretroviral therapy (HAART) severe complications of AIDS are rarely seen by newer generations of physicians and are not typically thought of; (3) the difficulties in evaluating altered mental status and weakness in an intubated patient receiving sedation. In immunosuppressed patients on mechanical ventilation, early evaluation with LP should be considered when altered mental status and fever of unclear etiology are present.

## 1. Introduction

Cytomegalovirus (CMV) is a member of the* Herpesviridae family* and often causes a self-limiting infection in immunocompetent individuals [1], while it can cause more severe disease including colitis, pneumonitis, and less commonly encephalitis, in profoundly immunocompromised patients [2]. In HIV/AIDS CMV resulting in disease most commonly occurs when CD4+ counts are less than 50 cells/ml [3] and most frequently presents as retinitis or gastrointestinal disease (esophagitis, colitis), while encephalitis or radiculopathies are uncommon [4]. CNS imaging findings are nonspecific [5], and diagnosis is made by identifying CMV in cerebral spinal fluid through PCR testing or cell culture. Once identified, antiviral therapy, with ganciclovir, foscarnet, or cidofovir should be initiated [6]. Survival after CNS CMV disease is poor [4, 6], with most data reported before anti-retroviral therapy.

## 2. Case Report

A 65-year-old male with hypertension and atrial fibrillation was admitted to the University of Rochester Medical Center with fever, chest pain, and dyspnea. A CT Chest angiogram revealed bilateral ground-glass opacities with mediastinal lymphadenopathy and no embolic disease. He was admitted to the general medicine service and was treated for community-acquired pneumonia with ceftriaxone and doxycycline. His fevers persisted for the first three days. On hospital day 6 his antimicrobial therapy was broadened to vancomycin, piperacillin-tazobactam, and azithromycin due to worsening hypoxia and was continued for 10 days. He was found to be HIV positive with a RNA level greater than 500,000 copies/ml and a CD4 count of 15. On hospital day 9 he required intubation for worsening hypoxia. He underwent bronchoscopy with bronchoalveolar lavage (BAL) with* pneumocystis jiroveci* identified on PCR testing and microscopy and CMV was identified on viral cell culture. Sulfamethoxazole/trimethoprim and glucocorticoid therapy was empirically started and he completed 21 days of therapy.

**Figure 1:**
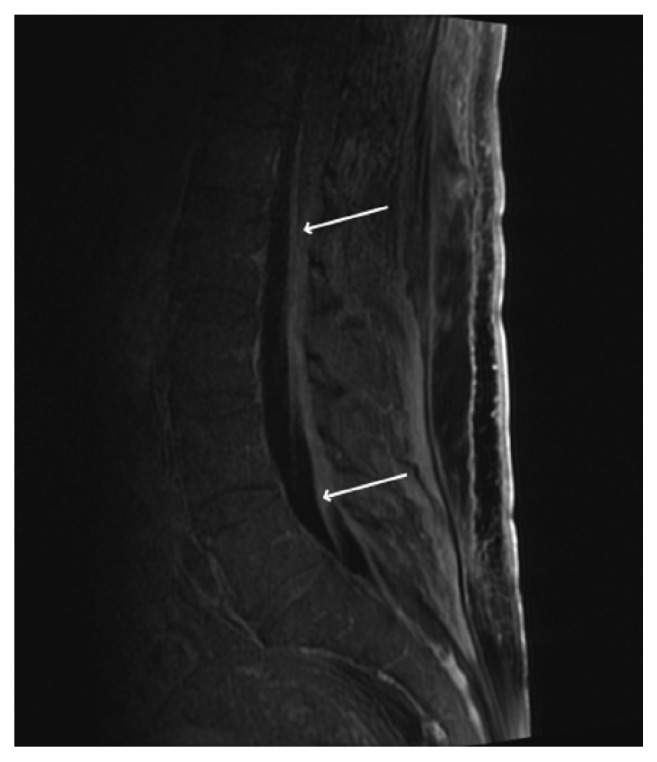
Sagittal view of MR lumbar spine T1 FLAIR depicting significant leptomeningeal enhancement and thickening of nerve roots (arrows).

On hospital day 23 he was extubated. Due to increasing lethargy he was reintubated on hospital day 27 for airway protection. After intubation he developed intermittent fevers for 25 days with altered mental status. Vancomycin and piperacillin-tazobactam were restarted. An initial work up for encephalopathy was performed with normal ammonia (18 *μ*mol/L), normal CT head with and without contrast, and negative evaluation for infection including blood, urine, tracheal aspirate, and stool cultures. An electroencephalogram was performed and showed moderate encephalopathy without epileptiform abnormalities. Highly active antiretroviral therapy (HAART) with elvitegravir, cobicistat, emtricitabine, and tenofovir alafenamide was started on hospital day 33 after HIV genotype testing returned. There was concern for drug fever from dexmedetomidine and piperacillin-tazobactam and both drugs were discontinued on hospital day 32 and 37. After no improvement in fevers a MRI of the head with and without contrast was performed on hospital day 37 and showed acute infarcts in the bilateral thalami, right parietal lobe, and right basal ganglia. Neurology felt it was embolic phenomena from atrial fibrillation. He also developed persistent, nonbloody loose stools with negative bacterial and parasite stool studies so serum CMV PCR was checked on hospital day 43 showing 2,623,108 IU/mL. With new nuchal rigidity on exam and right lower extremity weakness a lumbar puncture was performed on hospital day 44 after another head CT was normal. Spinal fluid analysis was consistent with encephalitis and CSF PCR for CMV DNA was positive (Table 1). After serum CMV PCR positivity returned ganciclovir was started on hospital day 47. MRI imaging of the cervical, thoracic, and lumbar spine showed extensive linear leptomeningeal enhancement along the lower spinal cord, conus medullaris, and roots of the cauda equine with thickening of the roots concerning for radiculomyelitis (Figure 1). At this point his mental status remained poor with quadriparesis and flaccid paraplegia. He underwent tracheostomy on hospital day 50.

Serum CMV DNA PCR and HIV RNA PCR were checked weekly to monitor treatment efficacy, and both decreased significantly on treatment (Table 2). No further lumbar punctures were performed. He received 30 days of IV ganciclovir 469mg twice daily before decreasing to IV ganciclovir 469mg daily after two consecutive weeks of undetectable serum CMV DNA PCR, hospital day 76. He received IV ganciclovir daily for 85 days total, before switching to oral valganciclovir 900mg daily. The serum HIV RNA PCR continued to decline and was 49 copies/mL on hospital day 128 (96 days after initiation of HAART therapy). He remained on mechanical ventilation and was able to move his upper extremities with assistance and could answer simple questions. He never regained function in his lower extremities. After minimal improvement he opted to stop mechanical ventilation and passed away on hospital day 147.

## 3. Discussion

This case report highlights the uncommon and rare presentation of CMV encephalitis and radiculitis, the limited data on treatment and prognosis in the HAART era, and the difficulties in evaluating altered mental status, fever, and extremity weakness in an intubated immunosuppressed patient.

CMV infection usually causes asymptomatic disease in immunocompetent individuals [1]. Severe CMV disease (colitis, pneumonitis, and encephalitis) is typically seen in profound immunodeficiency states including solid organ transplantation [2], bone marrow transplantation [7], and advanced AIDS [8] with CD4 counts less than 50 cells/*μ*L [3]. Although plasma quantitative CMV PCR is commonly measured in immunocompromised patients, its role in establishing CMV end-organ damage is unclear. The majority of individuals are exposed to CMV in their lifetime [4] and therefore testing for CMV PCR in peripheral blood is not always indicative of disease activity or severity [5]. According to the most recent NIH and CDC guidelines, blood assays to detect CMV DNA or antigen are not recommended for diagnosis of CMV end-organ disease because of their poor positive predictive value. CMV neurologic disease is diagnosed on the basis of a compatible clinical syndrome and the presence of CMV in CSF or brain tissue, most often evaluated with PCR. Initiating anti-CMV therapy should therefore be reserved until definitive diagnosis is established [6]. CMV encephalitis in AIDS is extremely uncommon with identification rates of ~2% of patients prior to the advent of HAART therapy [9]. With a decrease in AIDS, opportunistic infections are not frequently encountered by younger physicians and are thought of less commonly.

Disseminated CMV in the central nervous system can present with meningitis, encephalitis, or radiculopathies. Symptoms typically include altered mental status, delirium, confusion, weakness, or urinary retention [10]. Imaging with CT and MRI can have variable findings and in some instances can be normal [11]. Currently, diagnosis is made by identifying CMV in cerebral spinal fluid with PCR testing [12, 13]. Arribas et al. reported that AIDS patients with CSF CMV DNA molecules greater than 10^3^ per 8 *μ*L had severe CMV disease [14]. Once identified, treatment should be initiated, though the regimen of choice is controversial because there are no prospective trials evaluating antiviral therapy for CMV encephalitis. Given the poor outcomes in many patients with CMV-related neurologic disease, some experts recommend initiation of both IV ganciclovir and IV foscarnet [6]; however, there are significant toxicities associated with these medications (anemia, neutropenia, thrombocytopenia, nausea, diarrhea and renal dysfunction) and thus benefits and risks of therapy must be considered. The recommendation for dual therapy has been extrapolated from a randomized trial that found patients with relapsed CMV retinitis had better outcomes with the use of dual therapy as compared to those who received monotherapy with either of these agents [15]. Cidofovir has also been used [12]. Although immune reconstitution inflammatory syndrome (IRIS) causing worsening neurologic disease is a concern the optimal timing of starting HAART in AIDS patients with CMV encephalitis is unknown and has not been studied. Given the poor prognosis even in treated patients, therapy should be started as soon as the diagnosis is made. Time to clinical improvement without HAART is variable and can take as long as two months after starting antiviral therapy [16]. Duration of induction therapy varies between 2-6 weeks and should be continued until neurologic and virologic improvement is seen, after which maintenance therapy is initiated. Survival after diagnosis of neurologic CMV is poor [10]. Prior to HAART the median survival was 4.6 weeks from time of symptom onset [17] and in hospital mortality 38% [18]. There is no data on CMV encephalitis outcomes and management strategies in the HAART era.

Altered mental status/delirium is a common finding in patients in the intensive care unit and associated with increase hospital stay and mortality [19]. There are multiple etiologies including neurologic (stroke or seizure), infectious (sepsis, meningitis, encephalitis), temperature dysregulation (hyperthermia/hypothermia), metabolic (renal or liver dysfunction), medication (benzodiazepine), withdrawal (alcohol), hypoxia/hypercarbia, heart failure, or delirium [20]. Up to 80% of mechanically vented patients experience delirium [20]. Typically, a noncontrasted head CT is performed and abnormalities are found only a quarter of the time [21]. Infectious etiologies outside of the broad culturing (tracheal aspirate, blood, and urine culture) should always be considered in the work up of alerted mental status in an intubated immunosuppressed patient including lumbar puncture.

In our case report, we discuss the unique presentation of a patient with AIDS initially presenting with pneumocystis pneumonia and subsequently diagnosed with CMV encephalitis and radiculitis 6 weeks into his hospital stay after having persistent mental status changes and weakness while on mechanical ventilation. In the intubated immunosuppressed patient, broad differentials should be considered, including infectious etiologies, in patients with persistent altered mental status, fever, or weakness. Early recognition and initiation of therapy are key in improving outcomes of CMV in the central nervous system.

## Figures and Tables

**Table 1:** Results from lumbar puncture.

CSF	Results
Color	Colorless

Nucleated Cells per *μ*L	70

RBC per *μ*L	20

Polymorphonuclear cells %	89

Lymphocytes %	7

Glucose (mg/dL)	96

Protein (mg/dL)	272

Aerobic Culture	No growth

Gram Stain	No organisms

Fungus Culture	No growth

CMV DNA PCR (IU/mL)	>1.88x10^8^

EBV DNA PCR (IU/mL)	5,100

Cryptococcal Antigen	Negative

HSV Types 1 & 2 (copies/mL)	6,900 (HSV 2)

Varicella Zoster DNA	Negative

West Nile Virus RNA	Negative

**Table 2:** PCR and CD4 count.

Hospital Day														
	8	9	43	51	54	56	63	69	70	76	77	84	87	130

CMV PCR			2,623,108	223,152		33,296	538		<137					

HIV PCR	563,644				390				139		172		166	49

CD4 Count		15				16		26		23		34		39

## References

[1] Schottstedt V., Blumel J., Burger R. (2010). Human cytomegalovirus (HCMV)-revised. *Transfusion Medicine and Hemotherapy*.

[2] Ramanan P., Razonable R. R. (2013). Cytomegalovirus infections in solid organ transplantation: a review. *Journal of Infection and Chemotherapy*.

[3] Jacobson M. A., O’Donnell J. J., Porteous D., Brodie H. R., Feigal D., Mills J. (1988). Retinal and gastrointestinal disease due to cytomegalovirus in patients with the acquired immune deficiency syndrome: prevalence, natural history, and response to ganciclovir therapy. *QJM: An International Journal of Medicine*.

[4] Staras S. A. S., Dollard S. C., Radford K. W., Flanders W. D., Pass R. F., Cannon M. J. (2006). Seroprevalence of cytomegalovirus infection in the United States, 1988–1994. *Clinical Infectious Diseases*.

[5] Zurlo J. J., O'Neill D., Polis M. A. (1993). Lack of clinical utility of cytomegalovirus blood and urine cultures in patients with HIV infection. *Annals of Internal Medicine*.

[6] (2017). Panel on opportunistic infections in HIV-infected adults and adolescents. *Guidelines for the Prevention and Treatment of Opportunistic Infections in HIV-Infected Adults and Adolescents: Recommendations from the Centers for Disease Control and Prevention, the National Institutes of Health, and the HIV MEdicine Association of the Infectious Diseases Society of America*.

[7] Bhat V., Joshi A., Sarode R., Chavan P. (2015). Cytomegalovirus infection in the bone marrow transplant patient. *World Journal of Transplantation*.

[8] Spector S. A., Hsia K., Crager M., Pilcher M., Cabral S., Stempien M. J. (1999). Cytomegalovirus (CMV) DNA load is an independent predictor of CMV disease and survival in advanced AIDS. *Journal of Virology*.

[9] Gallant J. E., Moore R. D., Richman D. D. (1992). Incidence and natural history of cytomegalovirus disease in patients with advanced human immunodeficiency virus disease treated with zidovudine. *The Journal of Infectious Diseases*.

[10] Anders H., Goebel F. D. (1999). Neurological manifestations of cytomegalovirus infection in the acquired immunodeficiency syndrome. *International Journal of STD & AIDS*.

[11] Smith A. B., Smirniotopoulos J. G., Rushing E. J. (2008). Central nervous system infections associated with human immunodeficiency virus infection: radiologicpathologic correlation. *RadioGraphics*.

[12] Maschke M., Kastrup O., Diener H.-C. (2002). CNS manifestations of cytomegalovirus infections: diagnosis and treatment. *CNS Drugs*.

[13] Steiner I., Budka H., Chaudhuri A. (2005). Viral encephalitis: a review of diagnostic methods and guidelines for management. *European Journal of Neurology*.

[14] Arribas J. R., Clifford D. B., Fichtenbaum C. J., Commins D. L., Powderly W. G., Storch G. A. (1995). Level of cytomegalovirus (CMV) DNA in cerebrospinal fluid of subjects with AIDS and CMV infection of the central nervous system. *The Journal of Infectious Diseases*.

[15] (1996). Combination foscarnet and ganciclovir therapy vs monotherapy for the treatment of relapsed cytomegalovirus retinitis in patients with AIDS. The cytomegalovirus retreatment trial. The studies of ocular complications of AIDS research group in collaboration with the AIDS clinical trials group. *Archives of Ophthalmology*.

[16] Kim Y. S., Hollander H. (1993). Polyradiculopathy due to cytomegalovirus: report of two cases in which improvement occurred after prolonged therapy and review of the literature. *Clinical Infectious Diseases*.

[17] Holland N. R., Power C., Mathews V. P., Glass J. D., Forman M., McArthur J. C. (1994). Cytomegalovirus encephalitis in acquired immunodeficiency syndrome (AIDS). *Neurology*.

[18] Silva C. A., Oliveira A. C., Vilas-Boas L., Fink M. C., Pannuti C. S., Vidal J. E. (2010). Neurologic cytomegalovirus complications in patients with AIDS: retrospective review of 13 cases and review of the literature. *Revista do Instituto de Medicina Tropical de São Paulo*.

[19] Bleck T. P., Smith M. C., Pierre-Louis S. J.-C., Jares J. J., Murray J., Hansen C. A. (1993). Neurologic complications of critical medical illnesses. *Critical Care Medicine*.

[20] Hayhurst C. J., Pandharipande P. P., Hughes C. G. (2016). Intensive care unit delirium: a review of diagnosis, prevention, and treatment. *Anesthesiology*.

[21] Chokshi F. H., Sadigh G., Carpenter W., Kang J., Duszak R., Khosa F. (2016). Altered mental status in ICU patients: diagnostic yield of noncontrast head CT for abnormal and communicable findings. *Critical Care Medicine*.

